# A Cross‐System Comparison of Dark Carbon Fixation in Coastal Sediments

**DOI:** 10.1029/2019GB006298

**Published:** 2020-02-19

**Authors:** Diana Vasquez‐Cardenas, Filip J. R. Meysman, Henricus T. S. Boschker

**Affiliations:** ^1^ Department of Biotechnology Delft University of Technology Delft The Netherlands; ^2^ Department of Biology University of Antwerp Antwerp Belgium

**Keywords:** chemoautotrophy, fatty acids, *Beggiatoa*, cable bacteria, salt marsh, bioturbation

## Abstract

Dark carbon fixation (DCF) by chemoautotrophic microorganisms can sustain food webs in the seafloor by local production of organic matter independent of photosynthesis. The process has received considerable attention in deep sea systems, such as hydrothermal vents, but the regulation, depth distribution, and global importance of coastal sedimentary DCF have not been systematically investigated. Here we surveyed eight coastal sediments by means of stable isotope probing (^13^C‐DIC) combined with bacterial biomarkers (phospholipid‐derived fatty acids) and compiled additional rates from literature into a global database. DCF rates in coastal sediments range from 0.07 to 36.30 mmol C m^−2^ day^−1^, and there is a linear relation between DCF and water depth. The CO_2_ fixation ratio (DCF/CO_2_ respired) also shows a trend with water depth, decreasing from 0.09 in nearshore environments to 0.04 in continental shelf sediments. Five types of depth distributions of chemoautotrophic activity are identified based on the mode of pore water transport (advective, bioturbated, and diffusive) and the dominant pathway of microbial sulfur oxidation. Extrapolated to the global coastal ocean, we estimate a DCF rate of 0.04 to 0.06 Pg C year^−1^, which is less than previous estimates based on indirect measurements (0.15 Pg C year^−1^), but remains substantially higher than the global DCF rate at deep sea hydrothermal vents (0.001–0.002 Pg C year^−1^).

## Key Points

- Coastal dark carbon fixation rates are highest in diffusion‐driven intertidal areas and lowest in permeable continental shelf sediments
- Pore water transport and microbial sulfur oxidation mechanisms determine five depth distribution models of dark carbon fixation in sediments
- The renewal of labile organic matter by dark carbon fixation is important in coastal carbon budgets

## 1 Introduction

Chemoautotrophic microorganisms obtain their metabolic https://en.wikipedia.org/wiki/Energy by the https://en.wikipedia.org/wiki/Oxidation of various reduced inorganic https://en.wikipedia.org/wiki/Electron_donor, such as ammonium, nitrite, ferrous iron, and sulfide, and they use this energy to synthesize organic molecules from dissolved inorganic https://en.wikipedia.org/wiki/Carbon_dioxide dark carbon fixation (DCF). Chemoautotrophic microbes hence typically thrive in redox gradient systems, that is, transitions between reduced and oxidized environments, such as the chemocline of water bodies or the oxic‐anoxic interface in marine sediments (Jørgensen, 1982; Labrenz et al., 2005). In terms of seafloor environments, deep sea hydrothermal vents form the most conspicuous ecosystems, as both symbiotic and free‐living chemoautotrophic microorganisms are the main primary producers, which obtain their energy from the oxidation of sulfide and other reduced compounds that are enriched in the vent fluids (Nakagawa & Takai, 2008). Globally, however, the total sulfide production by sulfate reduction in coastal marine sediments is roughly one order of magnitude higher than the sulfide output from hydrothermal vents (Howarth, 1984). Therefore, coastal sediments have a far greater potential for chemoautotrophy‐based primary production than deep sea ecosystems. Using simplified model parameterizations, Middelburg (2011) estimated that DCF in coastal sediments (0–200 m) account for 38% (0.29 Pg C year^−1^) of the global oceanic DCF (0.77 Pg C year^−1^), thus contributing more than euphotic (31%) and deeper (12%) water column environments in the ocean. Yet the prevalence and magnitude of DCF in coastal sediments remains poorly documented.

In coastal sediments, sulfate reduction is the main respiration pathway, accounting up to 90% of the organic matter mineralization (Soetaert et al., 1996), and produces a large sedimentary pool of reduced sulfur compounds, such as dissolved free sulfide, thiosulfate, elemental sulfur, iron monosulfide, and pyrite (Jørgensen & Nelson, 2004). Only a small fraction (<10%) of this reduced sulfur is buried into deeper horizons (Howarth, 1984), and so, a large amount of energy within the reduced sulfur reservoir remains available for chemoautotrophic microorganisms that catalyze the oxidation of these reduced sulfur compounds. The microbial biomass that is newly produced by DCF becomes available in the microbial food web and eventually adds to the pool of organic matter in the sediment (Tsutsumi et al., 2001; Vasquez‐Cardenas et al., 2016).

The importance of DCF in sedimentary carbon cycling can be assessed by evaluating the CO_2_ fixation ratio, that is, the ratio of the CO_2_ fixed via DCF in relation to the total CO_2_ released by the mineralization of organic matter. Based on crude electron balance considerations and using globally averaged parameter values, this ratio has been estimated to be ~7% for a “typical” coastal sediment (Howarth, 1984; Jørgensen & Nelson, 2004), although recently, it has been argued that DCF could account for more than 30% of coastal sediment carbon cycling, using a different parameterization for global respiration rates and microbial growth efficiencies (Middelburg, 2011). These diverging estimates partially reflect the paucity of empirical data on coastal sediment chemoautotrophy, as only a handful of studies have experimentally determined and compared benthic respiration rates (e.g., sedimentary CO_2_ production or O_2_ consumption as a proxy for the mineralization rate of organic matter) with DCF rates (Bauer et al., 1988; Boschker et al., 2014; Dyksma et al., 2016; Enoksson & Samuelsson, 1987; Lenk et al., 2011; Santoro et al., 2013; Thomsen & Kristensen, 1997; Vasquez‐Cardenas et al., 2015), and the available data have so far not been used to calculate global estimates. Moreover, there is great variability in both the areal rate and the depth distribution of DCF between sites and habitats (Boschker et al., 2014; Enoksson & Samuelsson, 1987; Lenk et al., 2011; Lipsewers et al., 2017; Thomsen & Kristensen, 1997; Vasquez‐Cardenas et al., 2015). Hence, the question arises as to which environmental factors drive DCF and thus the CO_2_ fixation ratio in coastal sediments? At present, only one study has addressed this issue experimentally and detected no significant relationship between DCF and heterotrophic bacterial production, sediment respiration rate, or organic matter content (Santoro et al., 2013). In the present study, we provide a systematic round‐up of the current knowledge on DCF in coastal sediments, by combining the available literature data with a set of newly collected field data, and estimate global DCF production in coastal sediments.

## 2 Materials and Methods

### 2.1 Study Sites and Observations

DCF was investigated at eight coastal sites that cover a range in water depth (0–53 m), porosity (0.3–0.8), organic carbon content (0.03–3.00%), and oxygen consumption rates (3–86 mmol O_2_ m^−2^ day^−1^; Table 1). Four intertidal sites Rattekaai salt marsh (RK), Zandkreek (ZK), Oosterschelde sand flat (OSF), and westerschelde mud flat (WMF) were surveyed in the Rhine‐Meuse‐Scheldt Delta area (The Netherlands), which represent distinct habitats (salt marsh, oyster reef, and sand flat) in terms of their depositional and biogeochemical regimes. In addition, four subtidal shelf sites were sampled along a transect off‐shore in the North Sea running from the Wadden island of Terschelling (Dutch coast) to the Dogger Bank in the central North Sea (NS.15, NS.13, NS.8, and NS.4). A detailed description of the sites, the sediment sampling, and the determination of sediment characteristics (porosity, total oxygen uptake [TOU], and dissolved oxygen uptake [DOU]) are provided in supporting information, Tables S1 and S2.

**Table 1:** List of Dark Carbon Fixation (DCF) Measurements in Coastal Areas From This Study and in Literature

Site	Code	Water depth	BGC regime	DCF	Surface DCF	αCO2	Reference
				mmol m^−2^ d^−1^			
Gullmar Fjord, SW Sweden	StnL	Nearshore	Bioturbated	4.8	0.33	0.22	Enoksson & Samuelsson, 1987, b
Marine lagoon Faellesstrand NE Fyn Island, Denmark (incubated sediment for 24 days)	I.MLF24	Nearshore	Advective	3.1	0.18	0.17	Thomsen & Kristensen, 1997, b
Janssand sandflat (upper flat), German Wadden Sea	JS06	Nearshore	Advective	3	0.32	~0.02	Lenk et al., 2011, b
Brackish lagoon, Brazil	Br1	Nearshore	NA	1.0 ± 0.4	NA	0.03	Santoro et al., 2013, b
	Br2		NA	1.4 ± 2.6	NA	0.01	
	Br3		NA	0.8 ± 0.2	NA	0.02	
Rattekaai Salt Marsh, (Oosterschelde) the Netherlands	RK05	Nearshore	Diffusive	5.5 ± 1.9	0.93	NA	Boschker et al., 2014, a
	RK06			36.3 ± 4.8	0.98	0.19	
Zandkreek (Oosterschelde), the Netherlands	ZK05	Nearshore (salt marsh)	Bioturbated	2.6 ± 0.3	0.68	NA	
	ZK07			2.9 ± 0.2	0.61	NA	
Marine Lake Grevelingen, the Netherlands (time series incubation experiment)	I.MLG1	Nearshore	Diffusive	1.6 ± 0.5	0.84	0.03	Vasquez‐Cardenas et al., 2015, a
	I.MLG9			9.6 ± 2.4	0.79	0.21	
	I.MLG13			10.9 ± 0.9	0.38	0.14	
	I.MLG12			7.3 ± 2.2	0.55	0.09	
Intertidal sand, France	CS	Nearshore	NA	0.38	NA	NA	Dyksma et al., 2016, c
	CA		NA	0.50			
	JS13		Advective	1.10			
Marine Lake Grevelingen, the Netherlands	MLG1m	Nearshore	Diffusive	3.1 ± 0.5	0.38	0.1	Lipsewers et al., 2017, a
	MLG2m			1.9 ± 0.1	0.74	0.07	
	MLG3m			1.4 ± 0.3	0.44	0.05	
	MLG1a			0.2 ± 0.07	0.72	NA	
	MLG2a			0.8 ± 0.3	0.59	0.06	
	MLG3a			1.1 ± 0.5	0.78	0.06	
Kobbefjord, Greenland	KF.jun	Continental	Bioturbated	0.5 ± 0.07	0.32	0.04	Vasquez‐Cardenas et al., 2018, a
	KF.sep			0.6 ± 0.04	0.2	0.05	
	KF.dec			0.4 ± 0.2	0.41	0.06	
	KF.may			0.08 ± 0.05	0.29	0.01	
Rattekaai Salt Marsh, (Oosterschelde) the Netherlands	RK11	Nearshore (salt marsh)	Diffusive	8.6 ± 2.6	0.75	0.11	This study^a^
Zandkreek (Oosterschelde), the Netherlands	ZK11	Nearshore	Bioturbated	2.5 ± 0.9	0.5	0.05	
Oosterschelde sand flat, the Netherlands	OSF	Nearshore	Bioturbated	1.5 ± 0.5	0.6	0.04	
Westerschelde mud flat (Kapellebank), the Netherlands	WMF	Nearshore	Bioturbated	1.8 ± 0.5	0.38	0.02	
SE Frisian Front (station 15) North Sea	NS.15	Nearshore	Bioturbated	0.9 ± 0.06	0.46	0.04	
Dutch coast (station 13) North Sea	NS.13	Nearshore	Advective	0.07 ± 0.02	0.46	0.01	
SE Dogger Bank (station 8) North Sea	NS.8	Nearshore	Advective	0.2 ± 0.09	0.19	0.03	
NW Dogger Bank (station 4) North Sea	NS.4	Continental	Advective	0.07 ± 0.02	0.4	0.02	

*Note*. Each site is classified by water depth (Nearshore: 0–50 m b.s.l., Continental: 51–200 m b.s.l.) and biogeochemical (BGC) regime (advective, bioturbated, and diffusive). DCF rates and the relative contribution of surface DCF (0–1 cm) are stated as well as the CO_2_ fixation ratio (αCO_2_ = DCF/TOU). More details can be found in Tables S1 and S2. Technique used to measure DCF in sediments: ^13^C PLFA‐SIP method, ^14^C‐Scintillography, and ^14^C‐Scintillography‐FISH‐FACS.

Abbreviations: BGC = biogeochemical, DCF = dark carbon fixation, NA = not applicable.

a ^13^C PLFA‐SIP method.

b ^14^C‐Scintillography.

c ^14^C‐Scintillography‐FISH‐FACS.

In addition to these new field data, the available literature data on DCF in coastal sediments were compiled, which provided 26 additional observations on 12 sites. An “observation” refers to the determination of the DCF rate at a particular site at a particular time; some sites hence have data at multiple time points. For these sites, we compiled the following parameters (if available): DCF rate, TOU rate, DOU rate, porosity (ϕ), organic carbon content, in situ temperature (T), oxygen penetration depth, and water depth (Table S2). The CO_2_ fixation ratio (α_CO2_) was calculated as the ratio of DCF over the organic matter mineralization rate (R_min_) using either TOU or DOU as a proxy for R_min_.

### 2.2 Dark Carbon Fixation Rates

Within the compiled dataset, DCF rates were estimated via two methods: (1) incorporation of ^13^C‐DIC in bacterial phospholipid‐derived fatty acids and (2) incorporation of ^14^C‐DIC in the total organic pool using scintillography (Table 1). The first method thus describes bacterial chemoautotrophic activity while the second includes both bacterial and archaeal chemoautotrophic activity.

In our study, we analyzed bacterial phospholipid‐derived fatty acids combined with ^13^C‐stable isotope probing (Boschker et al., 1998). For all sites, ^13^C‐bicarbonate was added to the pore water with the line injection method, adding label through vertically aligned side ports in the core liners (0.5‐cm apart; 100 μl of label per hole). Labeled cores were kept for 24 hr at in situ temperature (±2°C) in a darkened incubator to prevent phototrophic carbon fixation. Cores were incubated with in situ seawater, and the overlying water was continuously bubbled with air.

At the end of the incubation period, sediment cores were sectioned into intervals (slicing pattern was site‐dependent) to a maximum depth of 6 cm. ^13^C‐incorporation into phospholipid‐derived fatty acid for each sediment layer was analyzed by gas chromatography—isotope ratio mass spectrometry (GC‐IRMS, Thermo, Bremen, Germany) on an apolar analytical column (ZB5‐MS Phenomenex).

Total bacterial DCF (mmol C m^−2^ day^−1^) was calculated as the cumulative activity in all depth layers. A detailed description of the phospholipid‐derived fatty acids combined with ^13^C‐stable isotope probing analysis can be found in supporting information and detailed calculations are described in Boschker and Middelburg (2002), Boschker (2004), and Vasquez‐Cardenas et al. (2015).

## 3 Results and Discussion

### 3.1 Cross‐System Comparison

**Figure 1:**
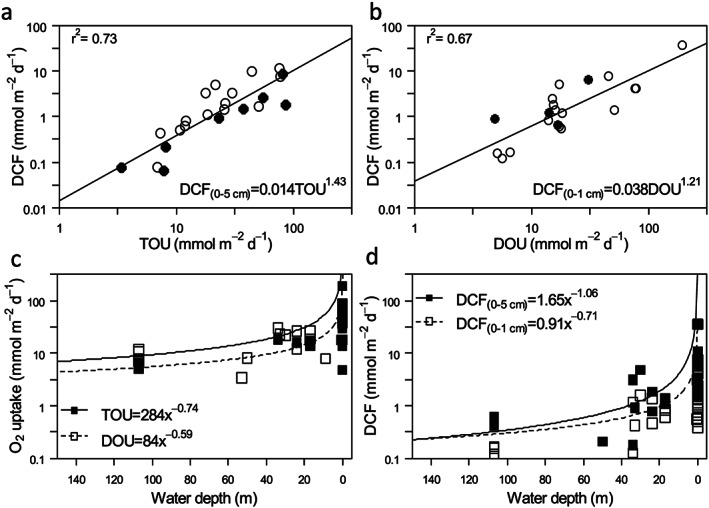
Comparison of dark carbon fixation (DCF) rates and oxygen uptake in coastal sediments. (a) DCF rates (0–5 cm) and the total oxygen uptake rate (TOU). (b) Surface DCF rates (0–1 cm) and the dissolved oxygen uptake rate (DOU). For plots (a and b), data from the present study are plotted in black, and data from literature are in white circles. (c) Regression model of sediment oxygen uptake at different water depth (Glud, 2008). (d) Regression models for DCF (μmol C m^−2^ day^−1^) in coastal sediments as a function of water depth (0–150 m). For plots (c and d), black squares are based on TOU rates and total DCF (0–5 cm), whereas white squares are based on DOU and surface DCF (0–1 cm) both from this study and from literature.

Our field survey revealed DCF rates varying from 0.07 to 8.60 mmol C m^−2^ d^−1^ (Table 1) and increased the number of available observations in coastal sediments by 30%. If we combine these data with existing literature data, the resulting cross‐system comparison shows that salt marsh creek sediments had the highest rates (36.3 mmol C m^−2^ day^−1^), while our North sea sediments (NS.4 and NS.13) exhibited the lowest rates (0.07 mmol C m^−2^ day^−1^). DCF rates hence vary widely, but up to present, only one study has looked into potential environmental drivers for this variability. When investigating 11 freshwater and brackish lakes from boreal and tropical locations, no significant correlation was found between area‐based DCF and sediment oxygen uptake, heterotrophic bacterial production (^3^H‐leucine incorporation), or organic carbon content (Santoro et al., 2013). Our cross‐system comparison also showed no significant correlation between DCF rates and organic carbon content (*r* = 0.26, *p* = 0.16, *n* = 30) or porosity (*r* = 0.17, *p* = 0.37, *n* = 32; supporting information, Figure S1). However, a strong correlation between sedimentary O_2_ uptake and DCF was apparent (Figures 1a and 1b). To this end, the DCF rates in the top centimeter (0–1 cm) of the sediment was compared to the DOU, assuming that reoxidation in the surface is fueled by O_2_ diffusion from the sediment‐water interface, while the depth‐integrated DCF rate was compared to the TOU, assuming that nonlocal injection of O_2_ at depth (e.g., by fauna burrow ventilation) can stimulate deep chemoautotrophic carbon fixation. Total DCF was significantly correlated to TOU (*r* = 0.87, *p* = 1.23 × 10^−7^, *n* = 23; Figure 1a) as was the surface DCF to DOU (*r* = 0.82, *p* = 6.693 × 10^−6^, *n* = 20; Figure 1b). Log‐log regression analysis of the DCF rates with either TOU or DOU provides a power law relation (*r* ^2^ = 0.73 for TOU and *r* ^2^ = 0.67 for DOU), which signifies that DCF is relatively more important in nearshore sediments, which typically display higher oxygen uptake rates (Glud, 2008). Additionally, the exponent of the power law between oxygen uptake and DCF exceeds one (DCF scales with TOU and DOU with an exponent of 1.43 and 1.21, respectively, Figures 1a and 1b), thus indicating that an increase in the oxygen consumption (as a proxy for organic matter mineralization) results in a more than proportional increase of DCF.

Sediments at greater water depths contain less labile organic matter than shallower sediments, because primary production tends to decline offshore as nutrients are supplied by terrestrial runoff, and organic matter is degraded more extensively the larger the distance it travels through the water column before reaching the sediment (Glud, 2008). To express the dependency of DCF on water depth more quantitatively, the power law relation between oxygen uptake rates and DCF can be combined with global regressions of oxygen consumption in marine sediments (Glud, 2008). Given the correlation between DCF and sedimentary O_2_ uptake (Figures 1a and 1b), and the finding that TOU and DOU scale with water depth with an exponent of −0.74 and −0.59, respectively (Figure 1c), the DCF rates thus scale with water depth with an exponent of −1.06 and −0.71, respectively (Figure 1d). Accordingly, we find that the sedimentary DCF is inversely related to the water depth within the coastal ocean.

### 3.2 Conceptual Biogeochemical Sediment Regimes

**Figure 2:**
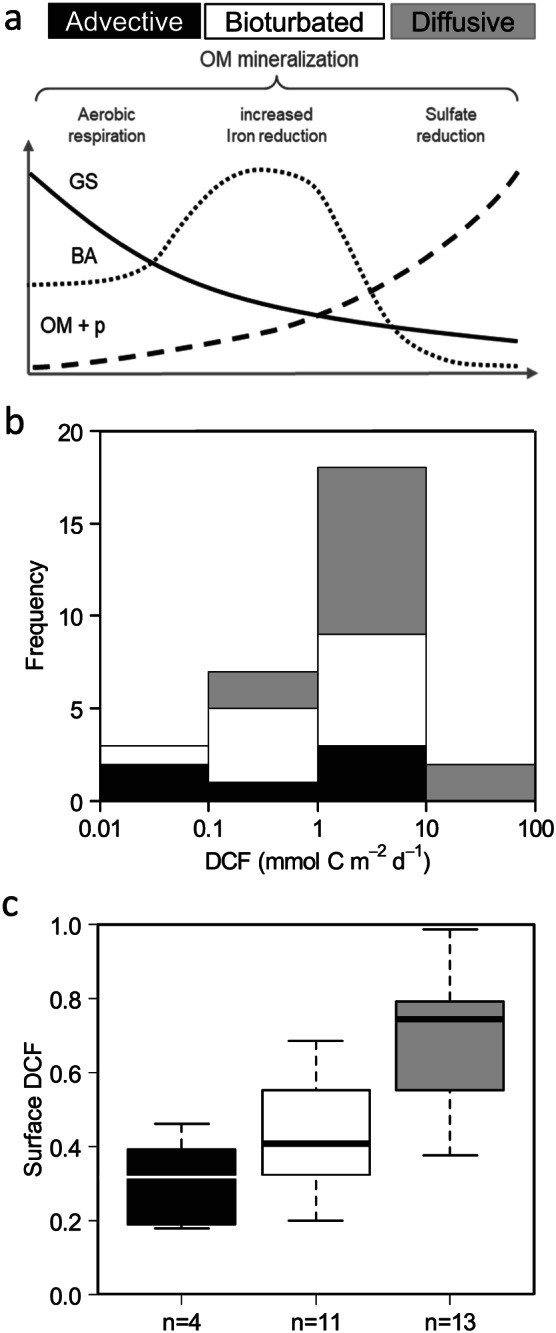
Dark carbon fixation (DCF) classified into different biogeochemical sediment regimes. (a) Classification of three biogeochemical sediment regimes: advective (black), bioturbated (white), and diffusive (grey). This classification takes into account grain size (GS), organic matter content (OM), porosity (p), and bioturbation activity (BA) in addition to the main mode of organic matter (OM) mineralization (aerobic respiration, iron reduction, and sulfate reduction). (b) The frequency of DCF rates for each biogeochemical sediment regime. (c) Boxplot of the relative contribution of surface (0–1 cm) to total DCF for the three biogeochemical sediment regimes.

DCF is principally enabled by reoxidation reactions between energetically favorable electron acceptors (O_2_, NO_3_^−^, and MnO) and suitable electron donors (H_2_S, FeS, FeS_2_, Fe^2+^, NH_4_^+^, and NO_2_^−^), and hence, we expect the transport mode of both electron acceptors and electron donors to be a key determinant of the magnitude and location of DCF in coastal sediments. As such, we propose a biogeochemistry‐oriented conceptual model of DCF in coastal sediments, which makes a distinction between three types of sediment regimes. These correspond to different modes of geochemical cycling based on the dominant physical transport regime that occurs in the sediment: *advective*, *bioturbated*, or *diffusive*. In general, the transport mode co‐varies with sediment characteristics (grain size and porosity) and the principal mode of organic matter mineralization (aerobic respiration, enhanced iron reduction, and sulfate reduction)—see review by Aller (2014; Figure 2a). All DCF rates were therefore categorized into one of the three sediment regimes based on porosity, organic matter content, and presence or absence of bioturbating fauna (Tables 1 and S2). When plotting the frequency of DCF rates for each sediment regime, advective, and bioturbated coastal sediments had consistently lower DCF rates than diffusive sediments (Figure 2b). To identify the different depth distributions of DCF associated to each sediment regime, we evaluated the relative contribution of DCF occurring at the sediment surface (0–1 cm) in relation to the total sediment activity (0–5 cm), and this was done for all sites where information is available (*n* = 29, Table 1). Diffusive sediments showed the highest relative contribution of DCF at the surface layer followed by bioturbated and advective sediments (Figure 2c and supporting information, Figure S2). Although these sediment regimes generate different interactions between electron donors and acceptors (as explained below), it should be noted that in reality, the depth distributions and the DCF rates are not always clear cut as sediments can be in transition between two biogeochemical regimes.

#### 3.2.1 Advective Sediments

**Figure 3:**
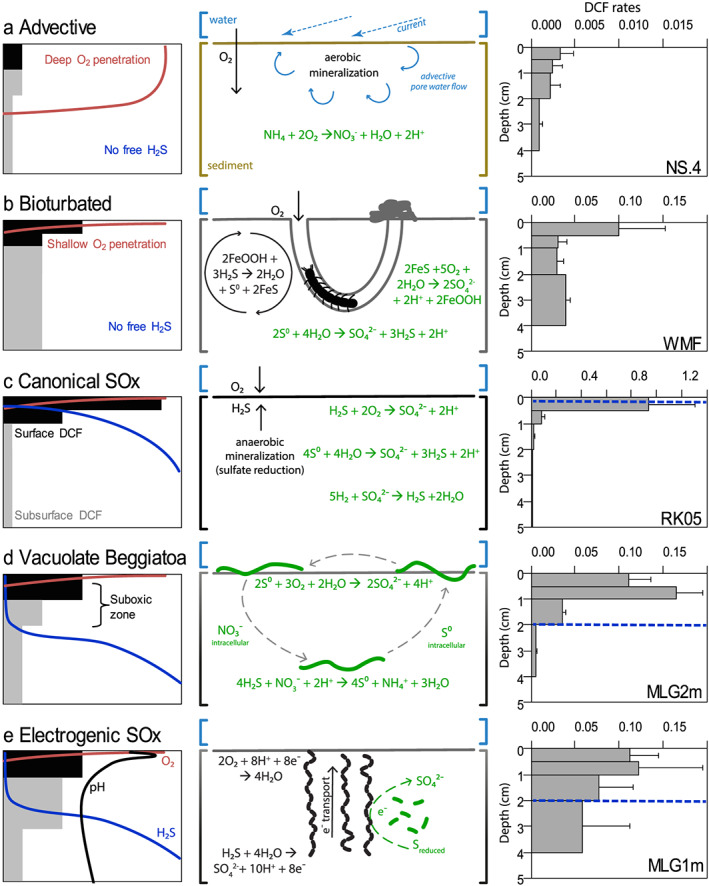
The depth distribution of dark carbon fixation (DCF) in five idealized biogeochemical regimes in coastal sediments. Left column: conceptual model of the depth distribution of DCF (black: surface activity, grey: subsurface activity) including the depth profiles of oxygen (red), free sulfide (blue), and pH (black) in pore water. Middle column: a schematic representation of the main biogeochemical reactions affecting the depth distribution of DCF in sediments. Green reactions indicate microbial activity associated to DCF. Right column: depth distribution of DCF rates (μmol C cm^−3^ day^−1^) as measured in diverse coastal sediments (top 5 cm); note the change in scale for DCF rates. Broken blue lines in plots (c–e) indicates the depth at which free sulfide was detected in pore water. Code for sites can be found in Table S2. (a) Advective‐driven sediments, mostly permeable, created by bottom water currents that produce deep oxygen penetration and high aerobic mineralization. (b) Bioturbated sediments where particle reworking and ventilation of burrow structures by fauna alter the reoxidation zones in the sediment. Black reaction denotes iron cycling by mixing of sediment particles by bioturbating fauna. (c) Canonical sulfur oxidation (SOx) in sediments with overlapping O_2_ and H_2_S. (d) Sulfur oxidation driven by intracellular redox shuttling by filamentous, motile, nitrate‐storing, colorless sulfur bacteria (*Beggiatoa*, green curved lines). (e) Hypothetical consortium occurring in electrogenic sediments between filamentous cable bacteria (green curved lines) and sulfur‐oxidizing chemoautotrophic bacteria (short green rods).

Permeable sediments consist of highly porous course‐grained sands, with low organic matter content and a relative low level of faunal reworking (Figure 2a). Physical advection, induced by waves and bottom currents, strongly influences the geochemistry, as the high permeability allows a deep intrusion of oxygenated bottom water in the sediment, thus favoring aerobic over anaerobic mineralization pathways (Huettel et al., 2014; Figure 3a). For example, the North Sea area studied (NS.4, NS.8, and NS.13) exhibits low sulfate reduction rates (up to 6.5 mmol C m^−2^ day^−1^; Upton et al., 1993) and a porosity lower than 0.40 (Table S2). Due to limited sulfate reduction and hence low production of sulfide in these sediments, ammonium and nitrite become the primary electron donors for chemoautotrophic activity via nitrification (Figure 3a). In the dark ocean (water column) and marine sediments, ammonia‐oxidizing archaea and nitrite‐oxidizing bacteria constitute key chemoautotrophic players that have a large influence in the nitrogen cycle (Guilini et al., 2010; Lipsewers et al., 2014; Pachiadaki et al., 2017; Reinthaler et al., 2010). However, nitrifying microorganisms typically exhibit low growth yields of approximately 0.10 (Bayer et al., 2019; Belser, 1984) and thus probably contribute minimally to the fixation of DIC in coastal sediments. Hence, low DCF rates (0.07–3.10 mmol C m^−2^ day^−1^, *n* = 6) with a small relative contribution of surface DCF (0.18–0.46, *n* = 5) characterize advective sediments (Figures 2b and 2c).

Note that in sediment core incubations, it is difficult to mimic the in situ advective transport of pore water, which may alter the depth distribution of DCF. The work by Thomsen and Kristensen (1997) is interesting in this respect as they incubated a permeable sandy sediment under laboratory conditions. As there was limited water flow in these incubations, pore water flow was probably low, mineralization was mainly by anaerobic processes, and therefore, DCF was relatively high for a permeable sediment (Table 1).

#### 3.2.2 Bioturbated Sediments

These sediments are typically found at intermediate porosities and organic matter levels and are inhabited by abundant macrofauna (Figure 2a). They are characterized by a shallow oxygen penetration and an absence of free sulfide in the pore water (Figure 3b), which are regulated by both bio‐mixing (solid phase transport) and bio‐irrigation (solute transport) by fauna (Kristensen, 1988; Kristensen et al., 2012; Meysman et al., 2006). During bio‐irrigation, the injection of overlying water into the sediment by macrofauna—for burrow ventilation or filter feeding purposes—provides a pulse of electron acceptors (O_2_ and NO_3_^−^) to the subsurface. This deep injection of electron acceptors can increase the decomposition of organic matter, but also stimulate DCF, such as nitrification along burrow structure (Kristensen & Kostka, 2005). Pore water rich in reduced compounds (e.g., elemental sulfur, thiosulfate, and hydrogen) is also pushed through the sediment during burrow flushing, which may stimulate anaerobic chemoautotrophic carbon fixation in subsurface sediment via S‐disproportionation or H_2_‐oxidization (Thomsen & Kristensen, 1997; Vasquez‐Cardenas et al., 2016). Additionally bio‐mixing, that is, particle reworking, strongly increases the iron cycling in the sediment, by stimulating the two‐way interconversion between iron sulfides (FeS) and iron oxides (FeOOH), whereby iron oxides are transported downwards and reduced, and iron sulfides are transported upwards and oxidized (Canfield et al., 1993; Kristensen & Kostka, 2005; Meysman et al., 2006; Seitaj et al., 2015). Consequently, a part of the mineralization will occur through dissimilatory iron reduction in addition to sulfate reduction (Figure 2a). The upward mixing of iron sulfides into the oxic zone potentially forms the main supply of reduced sulfur substrate for chemoautotrophs, which leads to a relatively high contribution (0.20 to 0.68) of surface DCF to the total DCF (Figures 2c and S2). The net effect of bioturbation on DCF is a stimulation of the total DCF activity (0.08–4.80 mmol C m^−2^ day^−1^, *n* = 11) in comparison to advective sediments (Figure 2b).

Within the dataset analyzed, seasonal changes in DCF depth distribution have been attributed to the seasonal decrease of bioturbation in sub‐Arctic sediments (Vasquez‐Cardenas et al., 2018). However, the biogeochemical effects of bioturbating fauna largely depend on the functional traits of the species present (Bertics & Ziebis, 2009; Laverock et al., 2010; Marinelli et al., 2002; Vasquez‐Cardenas et al., 2016). Therefore, two bioturbated sediments, with different species composition, may show different DCF depth‐distributions as seen among ZK11, OSF, WMF, and NS.15 (supporting information, Figure S3, fauna found at each site are described in supporting information).

#### 3.2.3 Diffusive Sediments

These sediments are cohesive with a low median grain size, high porosity, and high organic matter content (Figure 2a). They are characterized by high rates of sulfide production through sulfate reduction and, hence, typically display a high level of free sulfide in the pore water at depth (Figures 3c, 3d, and 3e). Diffusive sediments do not have large bioturbating fauna due to the toxic effects of sulfide on macrofauna (Rosenberg et al., 2002), and are neither impacted by physical advection due to their impermeability (Huettel et al., 2014), and thus are typically colonized by sulfur‐oxidizing microbes (Jørgensen & Revsbech, 1983; Wasmund et al., 2017). Often, the specific pathway of microbial sulfur oxidation can be inferred from depth water profiles of pH, H_2_S, and O_2_, as each microbial sulfur oxidation imposes a typical geochemical fingerprint upon the pore water (Seitaj et al., 2015). Consequently, diffusive sediments can therefore be further divided into three categories depending on the dominant mode of microbial sulfur oxidation present: canonical sulfur oxidation by microbes positioned at an overlapping O_2_‐H_2_S interface (e.g., *Thiobacillus*, *Thiovolum*, *Arcobacter*, non‐vacuolate *Beggiatoa*), sulfur oxidation by vacuolate *Beggiatoa*, and electrogenic sulfur oxidation (e‐SOx) by cable bacteria (candidatus *Electrothrix*). Highest DCF rates (0.20–36.30 mmol C m^−2^ d^−1^, *n* = 13), and surface DCF activity (0.38–0.98; Figures 2b and 2c) were identified in diffusive sediments due to the combined availability of free sulfide and favorable electron acceptors (O_2_ and NO_3_^−^).

##### 3.2.3.1 Canonical Sulfide Oxidation

In cohesive, organic‐rich sediments, where the organic loading is so high that reactive metal oxides are all reduced, free sulfide may accumulate in the pore water, and migrate upward to the sediment surface, where it comes into contact with O_2_ that diffuses from the overlying water into the sediment. This creates a narrow overlap between O_2_ and H_2_S, which is often only a tens of micrometers thick (Jørgensen & Nelson, 2004), and the sulfur oxidation that takes place is referred to as canonical sulfide oxidation (H_2_S + 2O_2_ ➔ SO_4_^2−^ + 2H^+^; Meysman et al., 2015; Figure 3c). The compiled dataset confirms that this diffusional reaction zone is a hotspot of sulfide oxidation associated with high surface DCF (Figure 3c and supporting information, Figure S3). DCF in deeper sediment horizons without O_2_ and NO_3_^−^ is much lower and is driven by S‐disproportionation (Bak & Pfennig, 1987; Vasquez‐Cardenas et al., 2017; Wasmund et al., 2017) and the oxidization of hydrogen through sulfate reduction (Miyatake et al., 2009; Thomsen & Kristensen, 1997; Wasmund et al., 2017). This DCF depth pattern linked to canonical sulfur oxidation was repeatedly observed in creek bed sediments of a salt marsh (RK05, RK06; Boschker et al., 2014) and at various stations in the sediments of marine Lake Grevelingen during summer hypoxia (MLG1a, MLG3a; Lipsewers et al., 2017). On some occasions (RK05, RK06, MLG2a) a white mat composed of filamentous bacteria that microscopically resembled non‐vacuolate sulfur‐oxidizing *Beggiatoa* was observed. At other times, no mat was notable at the O_2_‐H_2_S interface, and hence, DCF may have been carried out by colorless sulfur bacteria (e.g., *Arcobacter* and *Sulfurimonas*; Campbell et al., 2006). A similar DCF depth distribution was observed in the early phase of laboratory incubations, when sediment from marine Lake Grevelingen was homogenized and incubated with fully oxygenated overlying water (I.MLG1), where a narrow overlap between O_2_ and H_2_S was found (Vasquez‐Cardenas et al., 2015).

##### 3.2.3.2 Vacuolate *Beggiatoa*

Most coastal diffusive sediments generally exhibit a distinct separation between oxic and sulfidic horizons, a so‐called suboxic zone, where neither O_2_ nor H_2_S is present in detectable concentrations in pore water. “Gradient organisms” from the Beggiatoceae family (such as *Thioploca* and some *Beggiatoa*) can create such a suboxic zone because they are capable of intracellular redox shuttling between the oxic and sulfidic horizons (Jørgensen & Nelson, 2004; Sayama et al., 2005). These bacteria store nitrate intracellularly in a large central vacuole, and this reservoir is used at depth to oxidize free sulfide to elemental sulfur, which is also stored intracellularly (4H_2_S + NO^−^_3(int)_ + 2H^+^ ➔ 4S^0^_(int)_ + NH_4_^+^ + 3H_2_O). When migrating back to the surface, this elemental sulfur is further oxidized to sulfate in the presence of O_2_ or NO_3_^−^ (2S^0^_(int)_ + 3O_2_ + 2H_2_O ➔ 2SO_4_^2−^ + 4H^+^; (Preisler et al., 2007). A recent study of chemoautotrophic activity in sediments of a seasonally hypoxic marine lake, associated the presence of a thick suboxic zone (19 mm) with high densities of chemoautotrophic, vacuolate *Beggiatoa* in spring (MLG2m; Vasquez‐Cardenas et al., 2017). Furthermore, the depth distribution of DCF in the presence of *Beggiatoa* spanned from the surface to the sulfide appearance depth, in accordance with the sediment layer where *Beggiatoa* glided through. In contrast, in the case of canonical sulfur oxidation, DCF is limited to the oxic surface sediments. The observed decrease of DCF towards the sulfide horizon in MLG2m suggested that most of the DCF might be associated with the oxidation of elemental sulfur to sulfate rather than the oxidation of sulfide to elemental sulfur, but detailed physiological studies are needed to confirm this (Figure 3d).

##### 3.2.3.3 Electrogenic Sulfur Oxidation

e‐SOx has only been recently discovered and provides a second mechanism to create a suboxic zone in diffusive coastal sediments (Nielsen et al., 2010; Pfeffer et al., 2012). The process is performed by long filamentous bacteria, so‐called cable bacteria, which are capable of channeling electrons over centimeter‐distances along their longitudinal axis from cell to cell (Bjerg et al., 2018; Cornelissen et al., 2018; Meysman et al., 2019; Pfeffer et al., 2012). Although the physiological details of electron transport are currently unknown, the e‐SOx process involves two spatially segregated redox half‐reactions, that is, anodic sulfide oxidation, which occurs throughout the suboxic zone as well as in the top of the sulfidic zone (H_2_S + 4H_2_O ➔ SO_4_^2−^ + 10H^+^ + 8e^−^) and cathodic oxygen reduction, which occurs within the oxic zone (2O_2_ + 8H^+^ + 8e^−^ ➔ 4H_2_O). This proton production at depth and the proton consumption at the sediment surface induce a specific pH fingerprint upon the pore water, which serves to distinguish e‐SOx from other sulfur‐oxidizing mechanisms (Meysman et al., 2015; Seitaj et al., 2015; Vasquez‐Cardenas et al., 2017; Figure 3e).

The DCF depth profile associated with e‐SOx is highly remarkable, (1) as substantial DCF can occur deep down in the sediment, where electron acceptors such as O_2_ and NO_3_^‐^ are absent, (2) the DCF deepens as the sulfide front is pushed down by the development of the cable bacteria network, and (3) the centimeter‐deep DCF activity is tightly linked to the availability of oxygen at the sediment surface (Vasquez‐Cardenas et al., 2015). Based on their genome, cable bacteria are capable of DCF via de Wood‐Ljungdahl pathway (Kjeldsen et al., 2019) which would explain the documented chemoautotrophic carbon fixation at depth. However, in the initial study by Vasquez‐Cardenas et al. (2015) the strong DCF could not be attributed directly to the cable bacteria, but instead was likely performed by sulfur‐oxidizing Campylobacterales and Gammaproteobacteria. Whether the deep DCF thus occurs solely via cable bacteria, or is the result of a sulfur‐oxidizing consortium, remains to be determined.

In our survey of intertidal sediments, the e‐SOx geochemical fingerprint was observed, and cable bacteria were identified in the creek bed of salt marsh sediment in summer 2011 (RK11; Malkin et al., 2014). This was accompanied by high DCF from the surface to below the sulfide horizon (~15‐mm deep, supporting information, Figure S3). Similarly, the same DCF depth‐distribution was encountered in the seasonally hypoxic marine Lake Grevelingen, where also the e‐SOx geochemical fingerprint was observed hand‐in‐hand with high densities of cable bacteria (MLG1m and MLG3m; Lipsewers et al., 2017). These observations together confirm that high rates of DCF at depth are induced in coastal sediments by cable bacteria activity.

### 3.3 Contribution to Sedimentary Carbon Cycling

The newly synthesized microbial biomass via DCF largely drives the food web in deep sea environments such as hydrothermal vents (Nakagawa & Takai, 2008), while also in a shallow‐water hydrothermal system, DCF can account for more than 50% of the total DIC fixation by photo‐ and chemoautotrophy combined (Gomez‐Saez et al., 2017). However, in typical coastal sediments, the role of DCF in the carbon cycle is greatly understudied. The importance of DCF in coastal sedimentary carbon cycling can however be assessed by evaluating the CO_2_ fixation ratio (α_CO2_), for example, the ratio of the CO_2_ fixed via DCF in relation to the benthic carbon mineralization (α_CO2_ = DCF/R_min_). Note that the direct measurement of the benthic carbon mineralization is difficult, and so it is typically not recorded in marine sediments (and hence, R_min_ values are lacking in the dataset). However, the TOU is regarded as a suitable proxy for organic carbon mineralization rate in the sediment (Glud, 2008), and therefore, we can calculate the DCF/TOU ratio as a measure of α_CO2_. Across the dataset α_CO2_ ranged from 0.01 to 0.22, with a mean = 0.07 (*n* = 24), which corresponds well with the α_CO2_ = 0.07 for coastal sediments that is often cited in the literature (Jørgensen & Nelson, 2004). This latter value was derived for a “typical” coastal sediment based on the assumptions that (1) coastal DCF is dominated by sulfide oxidation, and other chemoautotrophic pathways can be ignored, (2) half the organic carbon is respired through sulfate reduction (0.50, accounting for the 1S:2C stoichiometry of sulfate reduction), (3) 90% of the sulfide produced via sulfate reduction is oxidized by sulfur‐oxidizing chemoautotrophs, and (4) sulfur oxidizers have a growth yield of 0.15.

**Figure 4:**
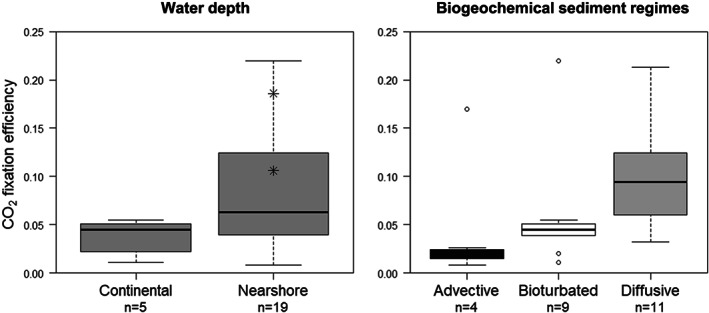
Boxplot of CO_2_ fixation ratio (α_CO2_) for two different coastal sediment zones based on water depth (left panel) and three biogeochemical sediment regimes (right panel). Only the α_CO2_ calculated from total DCF and TOU is shown. Values found in salt marsh sediment (Rattekaai, Nl) are denoted by (⁕). Number of samples (*n*) are specified in the *x*‐axis.

However, the broad range in α_CO2_ found in the dataset suggests that a mean value may not be the best way to determine the importance of DCF across the different systems studied. For instance, if we refer back to the water depth categories, then continental shelf sediments (51‐ to 200‐m water depth) have the lowest α_CO2_ = 0.04 ± 0.02 (mean ± standard deviation), while nearshore sediments (0 to 50‐m water depth, excluding brackish lagoons) have a α_CO2_ of 0.09 ± 0.07, and within this last zone, creek bed salt marsh sediments exhibit α_CO2_ of 0.11 and 0.19 (Figure 4). These values agree well with previous estimates by Howarth (1984) that were based on a much smaller dataset (providing 0.03–0.06 for continental shelf, 0.07–0.13 for nearshore sediments, and 0.10–0.18 for salt marshes). Moreover, if we estimate the mean α_CO2_ for the three biogeochemical sediment regimes, we find that advective sediments have the lowest values (0.02 ± 0.01, excluding the Thomsen and Kristensen observation), followed by bioturbated (0.06 ± 0.06) and diffusive sediments (0.10 ± 0.06; Figure 4).

This dependency on water depth and biogeochemical sediment regimes can be explained by evaluating the different components contributing to the α_CO2_. If we assume the DCF is governed by multiple reoxidation pathways *i*, then the CO_2_ fixation ratio can be expressed as

(1) $$\alpha_{\mathrm{CO}2}=\sum_{i}Y_{i}\lambda_{i}\gamma_{i},$$

whereby α_CO2_ scales as the product of three factors: (1) the production efficiency *γ*_*i*_, which scales the production rate of a given electron donor to the carbon mineralization rate; (2) the reoxidation efficiency*λ*_*i*_of the electron donor (i.e., how much electron donor is effectively used for microbial respiration and not diverted to abiotic oxidation); and (3) the CO_2_ fixation yield Y_i_ (e.g., the moles of CO_2_ fixed per mole of electron donor oxidized).

Creek bed salt marsh sediments fall within typical diffusive‐driven sediments, where sulfate reduction is the dominant respiration pathway (*γ*_*i*_ = 0.75–0.90), and nearly all sulfide is reoxidized by means of microbial metabolism (*λ*_*i*_ = 0.85–0.95; Howarth, 1984). Furthermore, high growth yields of aerobic sulfur‐oxidizers (Y_i_ = 0.16–0.56; Klatt & Polerecky, 2015) are expected in such sulfide‐rich environments. As a result, α_CO2_ is high in creek bed salt marsh sediments, and hence, DCF is a potentially underestimated source of labile organic matter in salt marsh and other active diffusive‐driven, nearshore sediments. At the opposite end, continental shelf sediments are often advective‐driven sediments that sustain sizeable mineralization rates via the catalytic bed filter effect (Huettel et al., 2014). The low α_CO2_ are caused by the centimeter‐deep penetration of oxygen through advective pore water flow (Huettel et al., 2003; Lohse et al., 1996), which favors aerobic mineralization of organic matter (*γ*_*i*_ << 1) and nitrifying bacteria with (generally) lower growth yields (*Y*_i_ < 0.1) than sulfur‐oxidizing bacteria. Furthermore, strong pore water flushing can flush electron donors out of the sediment thus preventing reoxidation in the sediment (low reoxidation efficiency *λ*_*i*_). Overall, the combination of these factors strongly diminishes the α_CO2_ in advective‐driven sediments (Figure 4).

### 3.4 Global DCF in the Coastal Ocean

The global DCF rate in the coastal ocean can be approximately estimated considering the sediment respiration of 0.82 Pg C year^−1^ (continental shelf: 0.29 Pg C year^−1^ and nearshore: 0.53 Pg C year^−1^; (Dunne et al., 2007). Based on the mean α_CO2_ for these two coastal depth zones (stated above), DCF would contribute an average of 0.01 and 0.05 Pg C year^−1^ in the continental shelf and in the nearshore, respectively. Estimations based on the biogeochemical sediment regimes are more problematic because the relative prevalence of these regimes is presently not well quantified. Yet advective and bioturbated sediments make up the majority of the seafloor of the coastal ocean. If we attribute 45% of the total benthic respiration (0.82 Pg C year^−1^) to advective sediments, 45% to bioturbated, and 10% to diffusive sediments, and we employ our estimated α_CO2_ for these three regimes, then advective sediments account for 0.01 Pg C year^−1^, bioturbated sediments for 0.02 Pg C year^−1^, and diffusive sediments for 0.01 Pg C year^−1^. This provides a total DCF of 0.04 Pg C year^−1^, which is 30% lower than our first estimate based on the water depth (0.06 Pg C year^−1^). Overall, our estimated DCF of 0.04–0.06 Pg C year^−1^ for coastal sediments (0–100 m) is two to six times lower than the previous estimates of DCF in nearshore (0.17 Pg C year^−1^) and shelf (0.12 Pg C year^−1^) sediments (Middelburg, 2011). It should be noted that these latter values were estimated indirectly, and hence carry more uncertainty, while our estimates are based on direct measurements of DCF. To put these DCF rates into a global context, open ocean DCF in the euphotic zone is estimated to vary between 0.24 and 11 Pg C year^−1^ (Baltar & Herndl, 2019; Middelburg, 2011) and between 0.10 and 0.30 Pg C year^−1^ in the dark ocean (Middelburg, 2011; Reinthaler et al., 2010). At deep sea vents, where chemoautotrophic production drives the food web, DCF rates are estimated at 0.001 Mg C year^−1^ to 0.002 Pg C year^−1^ (McNichol et al., 2018; Raven, 2009). Given the uncertainties in the underlying calculations and methods, further investigations are necessary to more precisely constrain the global DCF.

## 4 Conclusions

DCF varies broadly in coastal sediments related to mineralization rates, water depth, pore water transport mechanisms, and microbial sulfur oxidation pathways. DCF tends to be highest in diffusion‐driven intertidal sediments with high anaerobic mineralization activity and lowest in permeable continental shelf sediments driven by advective pore water transport and aerobic mineralization. In addition, we describe for the first time five distinct depth distribution patterns of DCF related to pore water transport and microbial sulfur oxidation mechanisms. Our global estimates of DCF based on α_CO2_ indicate that coastal sediments contribute between 0.04 to 0.06 Pg C year^−1^ to the oceanic carbon budget. However, more biogeochemical studies, including bacterial and archaeal DCF measurements within a diverse range of nearshore and shelf sediments, are necessary to consolidate the biogeochemical conceptual models proposed here and, hence, to better constrain the DCF production in the global coastal ocean. Similarly to the DCF occurring in the euphotic and dark open ocean, DCF in coastal sediments potentially offers a source of renewed labile organic matter that should be considered in food web analyses.

## Supporting information



Supporting Information S1

Table S1
