# Supplementary material for: A Cross‐System Comparison of Dark Carbon Fixation in Coastal Sediments
Source: Global Biogeochem Cycles. 2020 Feb 19;34(2):e2019GB006298. doi: 10.1029/2019GB006298 (PMC7375125; doi:10.1029/2019GB006298)
Supplement: Supplementary file 1 — Supporting Information S1 [file GBC-34-e2019GB006298-s001.docx]

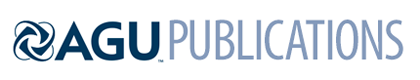


*Global Biogeochemical Cycles*

Supporting Information for

**A Cross-System Comparison of Dark Carbon Fixation in Coastal Sediments**

Diana Vasquez-Cardenas^1^, Filip J.R. Meysman^2,1^, Henricus T.S. Boschker^1,2^

^1^ Department of Biotechnology, Delft University of Technology, 2600 AA Delft, The Netherlands
^2^ Department of Biology, University of Antwerp, 2610 Wilrijk Antwerp, Belgium

**Contents of this file**

Text S1

Figures S1 to S3

Table S1

Caption for Table S2

**Additional Supporting Information (Files uploaded separately)**

Table S2

**Introduction**

This document contains a detailed description of the sampling sites and sample collection for the eight coastal sediments that were surveyed in this study. It also provides a more thorough description of the PLFA-SIP analysis and dark carbon fixation rate calculations. Techniques used to characterize the sediments and statistical analysis are listed. Supporting figures and tables can also be found.

Text S1. Supplementary Methods

**Sampling site description and sample collection**

Rattekaai (RK), is a salt marsh with cohesive sediment (porosity 0.74) with high organic matter loads (OC:3%) promoting high rates of sulfate reduction (Pallud & Van Cappellen, 2006). The sediment in the salt-marsh creek is generally black and sulfidic (i.e. detectable free sulfide in the pore water) up to the depth of oxygen penetration, and is often covered by a white mat of Beggiatoa-like sulfur-oxidizing bacteria, although Cable bacteria have also been recently found at the site (Malkin et al., 2014). The highest DCF up to now in literature were reported at the RK site (Henricus T S Boschker et al., 2014). Zandkreek (ZK) has a porosity of 0.54 and organic carbon content (OC) of 0.4%, that is characterized by Pacific oyster beds (Crassostrea gigas) that induce a high sedimentation of cohesive, organic rich material, which accumulates by biodeposition of the bivalves. Hence, the ZK site sustains high organic matter mineralization rates (Dauwe, Middelburg, & Herman, 2001) and can support significant DCF (Henricus T S Boschker et al., 2014). Still, the sediments are non-sulfidic (i.e. no free sulfide accumulates in the pore water) in the top 5 cm, and the sediments show high densities of macrofauna, thus indicating strong bioturbation (Henricus T S Boschker et al., 2014). The third intertidal site, the Oosterschelde Sand Flat (OSF) site is located in a tidal inlet, and supports medium sands with a porosity of 0.42 and OC of 0.2%. At the time of sampling, there was no free sulfide detected in the top 2.5 cm of sediment, and sediments were heavily bioturbated by high densities (> 50 m-2) of the lugworm Arenicola marina (Malkin et al., 2014). Finally, the Westerschelde Mud Flat (WMF) or Kapellebank, is a highly bioturbated site, characterized by benthic fauna such as Hydrobia, Macoma, Heteromastus, and Pygospio and high microphytobenthos densities in summer (Weerman, 2011); sediment had high silt content and free sulfide was not detected in the porewater. All four intertidal sites were visited between February and July 2011. Undisturbed sediment was collected during low tide using polycarbonate core liners (5 cm outer diameter and 20 cm in length; n=3), which was incubated to measure DCF. Large core liner (10 cm outer diameter and 30 cm in length) was used to collect intact sediment for total O2 consumption (TOU, n=3). A third set of cores liners (4 cm in diameter and 10 in length) were used to sample sediment for O2 microprofiling (n=3). Stations NS.4, NS.8 and NS.13 are categorized as non-depositional, in comparison station NS.15 on the Frisian front, which receives a higher organic matter input and has a higher silt content (Stoeck, Kröncke, Duineveld, & Palojärvi, 2002). Sediment was collected during a cruise onboard the RV Pelagia using a Reineck box corer. Subsequently polycarbonate core liners (5 cm outer diameter and 20 cm in length) were inserted into the box corer and used for chemoautotrophy measurements (n=3). A second set of cores (10 cm outer diameter and 30 cm in length) was also sampled from the box corer and was used for TOU measurements (n=3). No microprofiling was performed for these sites due to the high risk of breaking micro-sensors due the high content of shell fragments. Bioturbating macrofauna was observed at all stations. Fauna included sand tubeworms and brittle stars at the Dogger bank stations (NS.4 and NS.8), and sea urchins, razor clams, and polychaetes towards the Frisian front (NS.15) and near the Dutch coast (NS.13).

**PLFA-SIP analysis and dark carbon fixation rates**

A 20 mM stock solution of labelled NaHCO3 (99% 13C; Cambridge Isotope Laboratories, Andover, Ma, USA) was prepared in calcium- and magnesium-free artificial seawater in order to prevent carbonate precipitation. The stock solution was bubbled with N2 shortly before use to remove oxygen (thus preventing spurious reoxidation upon introduction in anoxic pore water). Two additional cores without 13C-label (per site) were used as controls and treated in the same manner as described in the methods.

At the end of the incubation period cores were sliced and sediment layers were collected in centrifuge tubes (50 ml), pore water was obtained by centrifugation (4500 rpm for 5 minutes) and sediments were lyophilized for PLFA-SIP analysis as in Guckert et al. (1985) and Boschker et al. (1998).

13C-incorporation was assumed to occur only via DCF as no 13C-incorporation was found in fatty acids typical for phototrophs (18:3ω3 and 20:5ω3) or eukaryotes (18:2ω6) indicating no direct or indirect 13C-uptake by these organism during the incubation (H T S Boschker & Middelburg, 2002). Incorporation rates of 13C into individual PLFA (expressed in μmol PLFA-C per g of dry sediment d-1) were calculated for each sediment layer as the product of individual bacterial fatty acid concentrations (μmol PLFA-C g-1) and its 13C fraction corrected for background values, divided by the incubation time. Incorporation rates were summed over all PLFA from 12:0 to 20:0 and converted to biomass carbon using a conversion factor of 55 mole biomass-C per mole of PLFA-C (Henricus T S Boschker et al., 2014). To arrive at volumetric DCF (i.e. μmol biomass-C per cm-3 of bulk sediment per day), rates were multiplied with the conversion factor ρ(1-ϕ), where ϕ is porosity and ρ is the solid sediment density; ρ was set equal to 2.60 g cm-3 for cohesive or 2.55 g cm-3 for permeable sediment. Rates were corrected for 13C-DIC enrichment in the pore water, which was measured by the head space technique with an elemental analyzer-IRMS equipped with a gas injection port (Henricus T S Boschker et al., 2014).

Note that anapleurotic reactions, i.e., CO2 fixation by heterotrophic bacteria utilized to replenish carbon intermediates in metabolic pathways, may account for 1.5% to 33% of the biomass production by heterotrophs (Wegener et al., 2012). However the PLFA-SIP method used here does not measure CO2 fixation through anapleurotic reactions, because the fixed CO2 by heterotrophic bacteria is not directly utilized in the synthesis of phospholipid fatty acids (Henricus T S Boschker et al., 2014). Accordingly, calculated DCF represent the “true” metabolism of chemoautotrophs.

**Sediment characteristics**

Porosity was determined from water content and wet density of sediment slices used for PLFA-SIP analysis. Lyophilized surface sediment was ground to a fine powder to determine the organic carbon content (OC) for each site using an elemental analyzer (Thermo Scientific; precision ±0.18%). To determine the total oxygen uptake (TOU) in sediments, cores were sealed with gas tight lids equipped with a central stirrer. Fiber optical oxygen sensors (FireSting OXF1100) were inserted through the lid and secured with rubber stoppers. TOU rates were determined from linear regression of oxygen concentrations in the overlying water versus incubation time. Incubations were stopped after a decrease of ~30% in O2 air saturation. Microsensor profiling of O2 on intertidal sediments was performed using micro-electrodes (Unisense A.S., Aarhus, Denmark) to obtain the oxygen penetration depth (OPD) and dissolved oxygen uptake (DOU) rates as described in Malkin et al. (2014). All parameters were measured in triplicates per station.

**Statistical analysis**

The eight observations on DCF obtained in the present study, in addition to the 26 observations reported in literature, as well as the associated environmental characteristics recorded, are listed in Table 1, S1 and S2. This dataset formed the basis of the statistical analysis. Pearson’s correlation coefficient (r) was calculated to investigate the linear dependence between DCF and several environmental parameters (porosity, OC, TOU, DOU). DCF and oxygen fluxes (DOU, TOU) were log transformed prior to linear regression analysis.


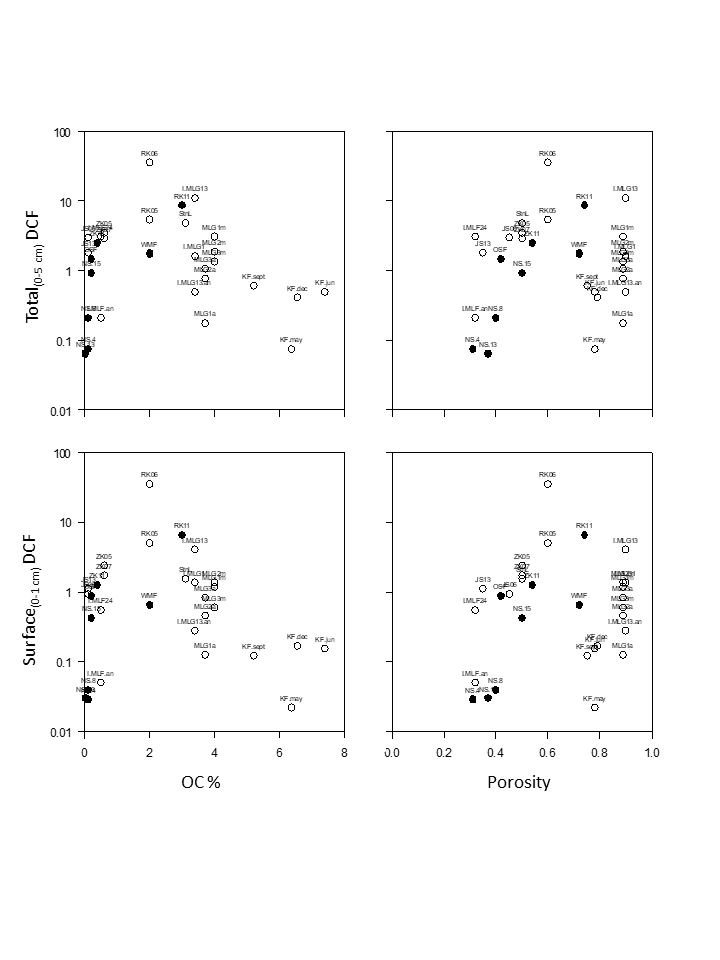
Figure S1. Total (top row) and surface (bottom row) dark carbon fixation (DCF) in relation to the percentage of organic carbon (OC) content (left column) and porosity (right column). Black circles are data from this study and white circles are from literature. Abbreviation of the site names can be found in Table 1 and Table S1.


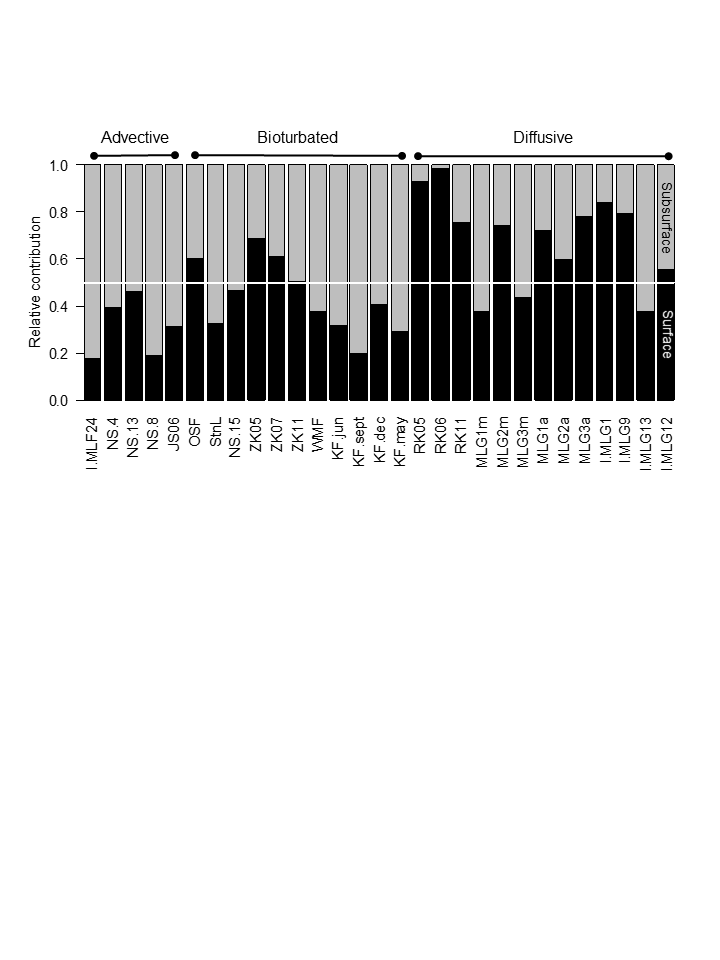


Figure S2. Detail of the surface (0-1 cm; black) to subsurface (1-5 cm; grey) DCF relative contribution for each observation of the data set. Sediments are organized (left to right) based on porosity in a increasing order (Table 1 and S1). The white horizontal line indicates an equal contribution of surface and subsurface activity. Abbreviations of sampling sites are listed in Table 1 and S1.


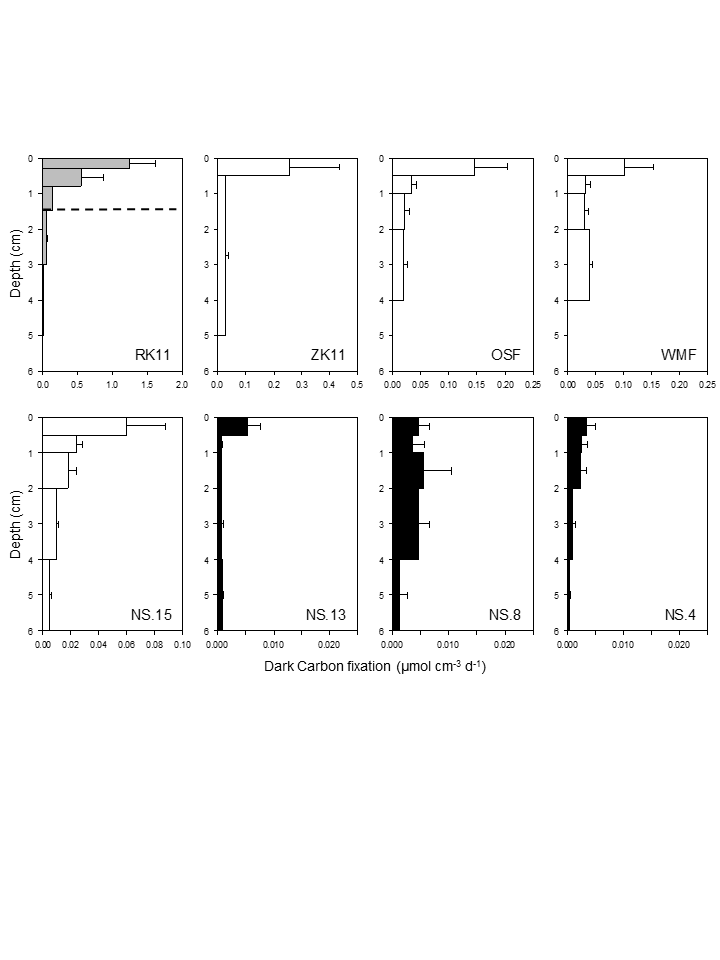


Figure S3. Figure S3. Different depth distribution of DCF from four intertidal sites in the Netherlands (top row), and four sites along a transect in the southern North Sea (bottom row). Abbreviation of the site names can be found in Table 1 and the color of the bars corresponds to the different sediment regime: grey = diffusive, white = bioturbated, black = advective. Note change of scale for DCF. The black broken line in RK11 indicates the depth at which free sulfide was detected in pore water, free sulfide was not detected in the other sites.

Table S1. Location and sampling date of the eight coastal sites from the present study where dark carbon fixation was evaluated. Other site characteristics can be found in Table S2.

Table S2. Physical, chemical and biological characteristics for dark carbon fixation (DCF) measurements in coastal areas from this study and in literature. Water depth (mbsl), porosity (ϕ), percentage of organic carbon content (OC%), and presence (P) or absence (A) of bioturbating macrofauna, biogeochemical (BGC) regime classification, maximum sediment depth studied, total (TOU) and dissolved oxygen uptake (DOU), oxygen penetration depth (OPD), and carbon fixation efficiency αCO2 based on total DCF and TOU as well as surface DCF (0-1 cm) and DOU.
